# From a learning opportunity to a conscious multidimensional change: a metasynthesis of transcultural learning experiences among nursing students

**DOI:** 10.1186/s12912-023-01521-4

**Published:** 2023-10-05

**Authors:** Juan M. Leyva-Moral, Betül Tosun, Rebeca Gómez-Ibáñez, Laura Navarrete, Ayla Yava, Mariela Aguayo-González, Ezgi Dirgar, Caterina Checa-Jiménez, M. Dolors Bernabeu-Tamayo

**Affiliations:** 1 Nursing Department, Faculty of Medicine, Universitat Autònoma de Barcelona, Avda. Can Domènech s/n, 08193 Bellaterra, Barcelona, Spain; 2 Nursing Department, Faculty of Health Sciences, University of Hasan Kalyoncu, Gaziantep, Turkey; 3 Consorci Corporació Sanitaria Parc Taulí, Barcelona, Spain; 4 Midwifery Department, Faculty of Health Sciences, University of Gaziantep, Gaziantep, Turkey

**Keywords:** Cultural competence, Metasynthesis, Nursing students, Qualitative methods, Transcultural

## Abstract

**Background:**

Several educational activities in nursing schools worldwide have been implemented to promote transcultural nursing and cultural competence. Despite the diversity of their experiences and outcomes, the available evidence has not been systematically reviewed and reinterpreted. This study aimed to review and reinterpret all rigorous qualitative evidence available, providing an opportunity to understand how students learn transcultural nursing and assisting faculties, researchers, managers, and practitioners in designing new interventions to improve transcultural training.

**Methods:**

A meta-synthesis was conducted to review and integrate qualitative studies of these phenomena. English, Spanish and Portuguese articles were searched in Pubmed and Scopus databases. Only peer-reviewed journals in which qualitative approaches were used were included. Quality was assessed using the CASP qualitative version checklist. The metasynthesis technique proposed by Noblit and Hare was used to analyse the data.

**Results:**

Twenty-nine studies were included in the analysis. Most studies used phenomenological approaches that were conducted in Australia and the United States of America, with international internships being the most popular learning method. The data revealed one central theme, “From learning opportunity to conscious multidimensional change,” and six subthemes. The transcultural nursing learning experience is not a simple or linear process. Instead, it appears to be a complex process formed by the interaction between a) self-awareness, b) reflective thinking, c) Cultural Encounters, d) cultural skills, e) Cultural Desire, and f) Cultural Knowledge.

**Conclusions:**

Transcultural nursing learning is a multifaceted process that arises from specific learning opportunities. This process is still to evolving. Therefore, specific educational strategies should be implemented to encourage attitudinal change and promote reflective thinking.

## Highlights

*What is already known:*

- Transcultural care promotes satisfaction among diverse populations.
- Cultural competence promotes an inclusive environment and mutual benefit.
- Cultural competence reduces biased decision making and promotes cultural awareness.

*What this paper adds:*

- Transcultural learning in nursing is a complex learning process.
- Transcultural nursing learning is multifactorial and has an impact on students' personal and professional lives.
- Transcultural nursing learning experiences involve self-awareness, reflective thinking, cultural encounters, cultural skills, cultural desires and cultural knowledge.

## Background

Transcultural nursing is defined as the academic study and practice of cultural beliefs, values, and lifestyles of diverse cultures, including the proper application of knowledge to provide culture-specific care to individuals, families, and groups of particular cultures; thus, through transcultural nursing, nurses can learn and explain how specific cultural care practices define well-being, death, or disabilities [1]. Consequently, nurses should become open-minded and flexible in caring for diverse populations, avoiding ethnocentrism by assessing and considering other cultures based on personal customs and values [2]. Ethnocentrism has been identified as a significant cause of negative intercultural sensitivity among nurses [3] and nursing students [4, 5]. Nursing students with high levels of ethnocentric attitudes showed less respect for cultural differences, were less responsive, confident or dedicated, and did not enjoy interacting with patients from different cultural backgrounds [5]. It is known that culturally competent nurses know other cultural practices and behaviors and can identify specific cultural patterns to provide individualized care to help meet patients' healthcare goals [6]. Therefore, culturally competent nurses understand cultural differences when providing care to diverse populations [7, 8]. Cultural competence has recently been defined as the continuous development of the ability and capacity to deliver secure and high-quality healthcare to patients from a variety of cultural backgrounds [9].

Evidence has shown several benefits of transcultural nursing practices. It extends beyond providing treatment or performing a procedure to promoting professional satisfaction and happiness and improving spiritual status [10]. Moreover, achieving cultural competency and transcultural nursing allows nurses to meet the needs of different groups of patients, develop efficient and realistic care interventions [11], and understand and accomplish an inclusive environment with mutual benefits and optimal care [12]. Similarly, culturally competent nurses are efficient in helping patients determine the best treatment option based on the patient context, family, socioeconomic level, and cultural background, which may lead to reduced health disparities [13]. In contrast, previous international research has identified several barriers to providing culturally competent care, including language barriers [14], work conflicts [15], reluctance [16], a wide range of cultural backgrounds of patients, a lack of resources, prejudices and biases of nurses [17], generational differences [17, 18], and lack of training [19, 20].

It has been observed in Europe that cultural competence is not clearly integrated in the nursing education and when it happens is somehow spontaneous without using a specific model [21, 22]. In this regard, it has been stated that stated that nursing faculty do not feel prepared enough to engage in culturally responsive teaching to meet the foreseen needs [23, 24]. Numerous programmes aiming to enhance nursing students' cultural competence have been established worldwide. The European Network of Nursing Education (ENNE) has organized an intensive one-week programme each year for over a decade, during which students and teachers from different European countries gather physically at one of the participating universities to receive training on topics linked to cultural competence and health. Student satisfaction with the programme is very favourable in terms of both content and teaching methodology [25]. The TC-Nurse project (https://tcnurse.eu/), funded by the European Commission, aimed to promote the development of social, civic and transcultural competences. The project demonstrated the importance of enhancing intercultural communication to avoid miscommunication and conflict between health professionals and patients [26]. The implementation of Content and Language Integrated Learning (CLIL) in educational interventions has shown positive results for participants in this type of programmes [21, 27]. In order to improve the intercultural education and training of nurses and allied health professionals across Europe, the European Commission funded project Intercultural Education of Nurses in Europe (http://ieneproject.eu/) was launched in 2008. Similarly, high levels of satisfaction and educational achievement were reported by nursing students who participated in the different training activities [28].

In Europe, there is a lack of integration of cultural competence in nursing education and, when it is present, it tends to be spontaneous rather than guided by a specific theoretical model [21, 22]. In this regard, it has been noted that nursing faculties do not feel prepared to implement culturally responsive teaching in order to meet the anticipated demands. Nursing schools and faculties have an important responsibility in instructing future nurses. Markey and Okantey [29] state that nurse educators influence the attitudes of their students by promoting positive attitudes towards intercultural inclusiveness. Furthermore, nurses can develop initiatives to increase cultural sensitivity and avoid stereotypes, bias and discriminating care practices. To address the aforementioned training gap, various activities such as simulation [30, 31], digital applications [32], online courses [33], intensive programmes abroad [25] and role-playing [34] have been implemented by nursing schools worldwide to enhance transcultural nursing and cultural competence. Tosun et al. [22] found that only ten studies demonstrated a statistically significant improvement in culture-related competences after implementing a diverse range of teaching strategies. Thus, assessing their impact on the learning of student nurses is a challenge due to the heterogeneity of results found in the previously mentioned studies. Unfortunately, nursing students' subjective personal experiences of transcultural nursing learning experiences have not been systematically reviewed and reinterpreted to gain a fuller understanding of these phenomena. Hence, the aim of this study is to review all available rigorous qualitative evidence and reinterpret the findings to gain a full understanding of nursing students' transcultural learning experiences. Thus, the aim of this study is to thoroughly review all available high quality qualitative evidence and reinterpret the findings to provide an in-depth understanding of nursing students' transcultural learning experiences. This could offer novel perspectives on how students acquire transcultural nursing knowledge and enable educators, researchers, managers, and practitioners to devise novel measures to enhance transcultural education.

## Methods

### Design

A metasynthesis was conducted better to understand the transcultural learning experiences of nursing students. A qualitative metasynthesis is a collection of qualitative results about a particular phenomenon from several research studies, the collective results of which may provide a better idea than the results of a single study [35, 36].

### Search methods

PubMed and Scopus databases were screened. The search strategy was guided according to the Preferred Reporting Items for Systematic Reviews and Meta-Analyses statement [37]. Transcultural, nursing, learning, and qualitative are examples of English keywords used and their translation into Spanish and Portuguese (see search examples in Table 1). Boolean operators AND, OR, NOT, and truncation tools were used for each database. The researchers were fluent in Portuguese, Spanish, and English; hence, the research was limited to peer-reviewed articles published in scientific journals between 2011 and 2022. Only qualitative studies or mixed methods studies reporting qualitative findings on transcultural learning experiences were included. Finally, studies using different methodologies and theoretical perspectives were included to increase the legitimacy and transferability of the findings. Studies scoring less than 60% in the CASPE Checklist were excluded.

**Table 1:** Search Strategy Example

Pubmed
(((((((((((transcultur*) AND (student*)) AND (qualitative))) AND ((((((((transcultur*) AND (student*)) AND (qualitative))) AND (learn*))) AND (internatio*)))) AND ((((((((transcultur*) AND (student*)) AND (qualitative))) AND (learn*))) AND (competenc*))))) AND (nursing studies)) OR (nursing degree)) OR (nursing bac*)) OR (diploma)) AND (undergrad*)) Limited to English & 10 years
Scopus
TITLE-ABS-KEY(transcult* AND nurs* AND learn* AND qualitative OR qualitatively OR qualitatives, transcult* AND nurs* AND learn* AND qualitative OR qualitatively OR qualitatives) AND PUBYEAR > 2010

### Quality appraisal

**Fig. 1:**
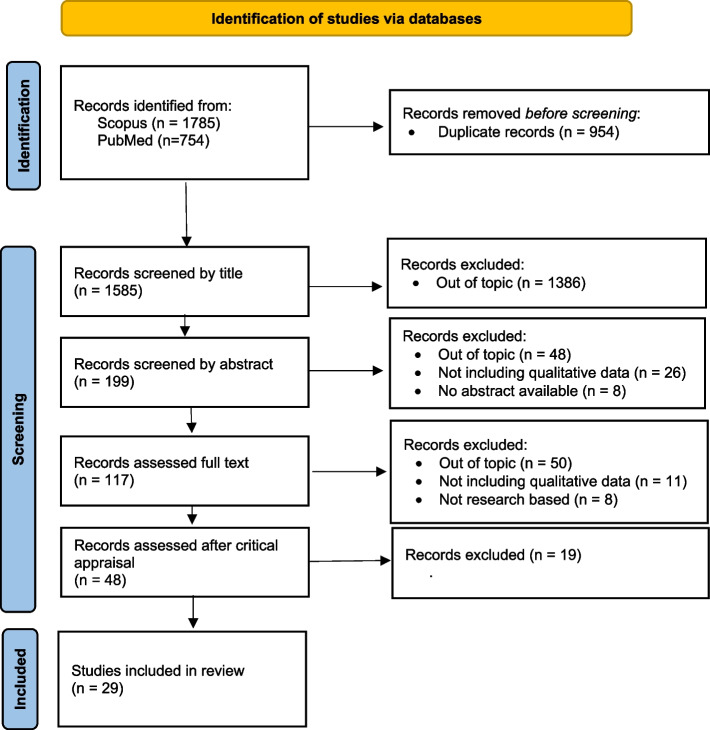
Prisma Flowchart

After deleting 954 duplicates, 1,585 studies were selected for further review. Four pairs of reviewers reviewed the titles, abstracts, and full text of the studies. Peer review aimed for 95% agreement; otherwise, the principal investigator made decisions as an external judge. The qualitative version of the Critical Appraisal Skills Programs [38] was used to assess quality (Fig. 1).

### Data synthesis

The data were analyzed using the method described by Noblit and Hare [39]. Data extraction for the study was conducted through a customized form in Microsoft Excel. First, the relationships, similarities, and dissonances between the studies were identified. Reading, viewing, comparing, and contrasting were mandatory. Next, key attributes and findings were summarized in an ad hoc table, with special emphasis placed on comprehending key metaphors, phrases, ideas, concepts, and relationships [39]. The analysis was conducted independently by the principal investigator (JL) and a research assistant (LN), and it was later reconciled in debriefing and confirmation meetings. During this process, key concepts and conceptual themes were identified. Themes, metaphors, and concepts from all included studies were thoroughly compared, with the original findings retained and the findings, concepts, and themes integrated into a collective metasynthesis. Redundancy not only ensured the validity of interpretations but also served to complement them. The focus was on the complexity, depth, richness, and meaning of concepts and their relationships [40]. The final diagram helped in conceptualizing the identified interpretations and themes. Finally, a debriefing session was held with experts in transcultural nursing and qualitative methods to confirm and verify the findings.

### Ethical consideration

Ethical approval was not required because all included studies had been previously approved by their respective institutional review boards.

## Results

After reading the full texts and assessing their quality, 29 studies were finally included in this metasynthesis. Most of the studies were led by north American [41–48], Australian [49–53], Canadian [54–58] and European universities [59–64]. International or culturally diverse clinical placements were the most frequent teaching method [43, 44, 46, 48–55, 57, 59, 61, 62, 65–68]. Most of the studies did not specify the qualitative method used [42, 45, 49–51, 53, 55, 63–65, 69]. Of the studies that reported the method used, phenomenology was the most common [46, 48, 52, 56, 58, 60–62, 68] Table 1 summarizes the main characteristics of the twenty-nine studies.

**Fig. 2:**
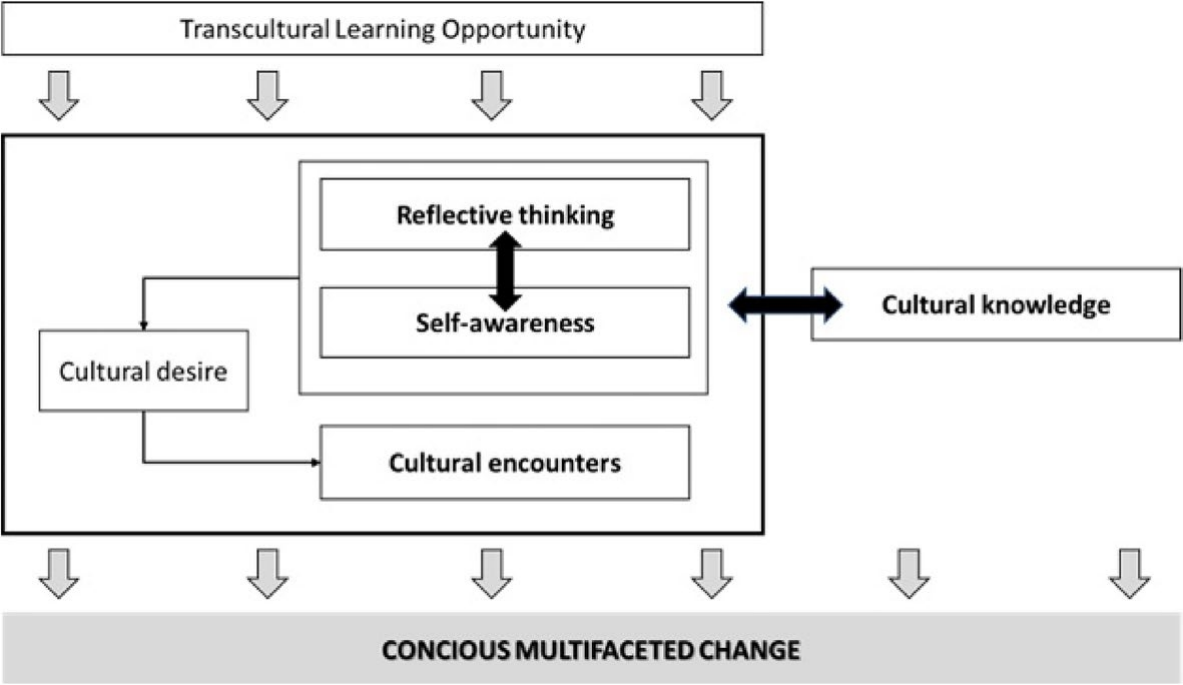
Graphical representation of the Transcultural Nursing Learning Experiences

The data showed one central theme (“From learning opportunity to conscious multidimensional change”) and six subthemes (Table 2). This helped in understanding transcultural nursing learning experiences as being somewhat beyond the scope of the practice itself. However, it is a complex learning process with many influencing factors. These significantly impact the personal and professional lives of the students (Fig. 2).

**Table 2:** Codes and Themes

MAIN THEME	THEMES	SUB-THEMES	CODES
From learning opportunity to conscious multidimensional change	Self-awareness	Gaining insight	Getting out of my comfort zone
			Culture influences my existence
			Self-awareness
			Egolessness
			Realizing the importance of public health education
			Life transforming experience
			You must live it to understand it
		Eyes-opening experience	Perceiving inequalities
			Embracing reality
			Awareness of cultural differences
			Understanding cultural differences
			Learning to respect beliefs
			Experiencing professional cultural differences
			Understanding family care
			Experiencing family care
			Health-threating infrastructures
			Awareness of other health problems
			Increasing cultural awareness
			Identifying diversity
			Experiencing helplessness
			Conscious use of sanitary material
		Identifying previous attitudes	Ethnocentrism
			Identifying own stereotypes
			Learning to deal with my own difficulties
			Focusing on me
			Cultural supremacy
			Racism
	Reflective thinking and practice		Self-awareness
			Identifying my own values
			Considering diversity
			Reflective practice
			Identifying benefits of transcultural care
			Self-reflection
			Personal growth
			Changing my way of thinking
			Debate opportunity
	Cultural Encounters	Ways of learning	Many ways of learning
			Mirroring
			Interaction
			Debriefing
			Active learning
			Experts
			Peer learning
			Practical learnings
			Understanding beliefs
			International discussions
			Role playing
			Webinars
			History
			Learning from diversity
			Humility
			Real interactions
			Extra curriculum learning
		Communication problems	Careless process without communication
			The importance of communication
			Communication barriers
			Communication skills
	Cultural skills	Lessons learned	How to deal with others' prejudices
			Breaking prejudices
			Promoting cultural sensitivity
			Identifying inappropriate behaviors
			Breaking cultural barriers
			Avoiding ethnocentrism
			Cultural self-efficacy
			Reducing racism
			Empowerment
			Improving cultural confidence
			Commitment
			Feeling ready to work with diverse populations
			Avoiding cultural blindness
			Accepting different treatments
			Culturally sensitive actions
			Understanding social justice
			Eliminating prejudices and stereotypes
		Being ready for diversity	Understanding privileges
			Understanding diversity
			Cultural sensitivity
			Promoting advocacy
			Respecting subjective beliefs
			Being used to difference
	Cultural Desire		Needing specific training
			Identifying my lack of cultural competence
			Cultural blindness
			Identifying non-effective caring practices
			Feeling non-sufficient
			Curiosity
			Need to improve cultural knowledge
			Insecurity
			Need to be culturally competent
	Cultural Knowledge		Understanding religious care practices
			Understanding family care
			Understanding cultural differences
			Understanding theoretical concepts
			Identifying social injustice
			Understanding cultural practices
			Gender issues
			Religion and spirituality

### From learning opportunity to conscious multidimensional change

The elements of transcultural nursing learning experiences are explained in this central theme. However, this is not a straightforward linear process. Instead, it appears to be a complex process resulting from the interaction of six themes, which gives meaning to the entire experience. These were the themes (a) self-awareness, (b) reflective thinking, (c) Cultural Encounters, (d) cultural skills, (e) Cultural Desire, and (f) Cultural Knowledge.

Transcultural nursing learning experiences do not appear to be spontaneous. These experiences are the result of faculty commitment, motivation, and interest. Therefore, promoting learning opportunities for transcultural nursing learning experiences is necessary. These opportunities do not necessarily imply living abroad for a while, even though activities abroad provide realism and meaningful learning that impacts the lives of nursing students. Several pedagogical approaches have been identified in the literature, such as webinars, expert lectures, role-playing, and mirroring.

### Self-awareness

Following Transcultural Nursing Learning Experiences implementation, reflective thinking is promoted and consolidated, providing access to a state of self-awareness that identifies identity, behavior, preferences, comfort, difficulties, feelings, and practices related to care and diversity. Moreover, this state of self-awareness activates reflective thinking, resulting in an attitudinal cycle that feeds back and continues to be active. Nursing students refer to this as a state of gaining insight, allowing them to eliminate their egoless attitudes. Students realize how important it is to step outside of their comfort zone to understand how culture influences their existence and presence in the world; therefore, they describe it as a life-changing experience that allows them to open their eyes and embrace reality. Self-awareness influences how inequalities are perceived, and they affect care. Students also understand the significance of recognizing and respecting cultural differences and family practices. Consequently, self-awareness fosters cultural awareness. Finally, self-awareness encourages identifying negative attitudes related to diversity, such as ethnocentrism, prejudices, stereotypes, white supremacy, and racism.

*I need to be aware of cultural differences and reflect on how that can help me understand my own culture or myself better. By mirroring myself in the light of others will help me care for my patients coming from a different culture* [63].

*I found myself getting defensive for them [Arab women] and trying to explain that they don’t really understand them. They really don’t understand how it is they are just going by assumptions. I have direct contact with these women, and I can see that they are in wonderful, loving family relationships. I think [participating in this program] definitely did change my perception* [45].

*I think that it [educational activity] showed how much of a throwaway society we are in Australia compared to them [Nepal] they are using everything they have got available and whereas over here, we drop a pair of gloves on the ground, and we just throw it in the bin. Whereas over there, that is something that they would reuse as a tourniquet or something else* [51]*.*

### Reflective thinking

All transcultural nursing learning experiences were based on reflective thinking and practice. These types of activities helped students to identify their values and beliefs. In addition, by observing, reflecting, discussing and understanding how others behave according to their local cultural practices, students were able to see the benefits of practicing transcultural nursing. Therefore, self-reflective practices promoted cultural self-awareness and helped students to consider diversity in their nursing practice.

*I need to be aware of cultural differences and reflect on how that can help me understand my own culture or myself better. By mirroring myself in the light of others will help me care for my patients coming from a different culture* [63].

*I work in addiction and mental health as my background, and I questioned myself when visiting the addictions program. That is not how we treat our patients in Canada. We do not treat with love; it is you are guilty until you are clean again. I saw a change within myself because I like to think I do not judge, and I want to treat people with the respect they deserve* [58].

### Cultural encounters

Exposure to and interaction with diverse populations promotes significant transcultural learning. Nursing students describe different learning experiences as a result of their cultural encounters. These activities were all designed and implemented as extracurricular activities. Most of the teaching approaches, in line with reflective learning, put students at the centre of the action. Thus, active learning methods were implemented with significant results based on the students' narratives. These teaching activities include mirroring, debriefing, peer learning and role-playing. Through meaningful cultural encounters, students ultimately understand the importance of humility in appreciating the beliefs and practices of others and learning how to respect and implement them. These cultural encounters also help students to understand the historical and socio-political issues surrounding cultural practices. Finally, it should be noted that these transcultural encounters do not happen by chance. To be meaningful for their learning, they require a cultural desire, attraction or willingness to care for those who are different from the students.

*The discussion with foreign peers also enriched my thinking and planning, they raised out points that I didn't think of and led me to think in a more comprehensive way* [63].

*This international collaboration has been one of the most valuable experiences I have been part of. My ideas about nursing care have been constantly challenged and has made me question the protocols we have in Australia* [64].

*I don’t completely understand English, but I was proud of my English. However, I was shocked because there were many cases when I misunderstood what the patients were saying. English or the Language of that country … speaking the native language is very important to become a global leader for nurses* [67].

### Cultural skills

This theme refers to the skills gained through cultural competence, interactions, and reflective thinking. Students say that the more cultural skills they learn, the better the culturally competent care they provide. In addition, these cultural skills enable nursing students to recognize, understand, appreciate, and respect issues previously unconsidered when caring for a patient. Finally, cultural skills enabled nursing students to develop person-centered, useful, and meaningful nursing care plans that considered the real needs of the patients.

*It [the clinical experience abroad] made us more culturally competent and more confident in dealing with different people in Oman who come from different educational levels and from different regions as they have same beliefs as us but they also have different beliefs. This experience helped me feel more confident to deal with all and not to make decisions for a person from the outside [perspective], out of fear* [45]*.*

*It was interesting because straight away I was like trying to put myself in the person’s position but there were a few others in class that just couldn’t understand the cultural side of it. I think they found it difficult because they had never experienced something that culturally different before. Obviously, having just been in Nepal, I was like I know this* [51]*.*

*During the course, especially from the role play, I realized that I may harm immigrant mothers because of my neglect. Through the course, I realized that I should be patient and careful with cultural differences while communicating with immigrants. Otherwise, we could hurt their feelings* [66]*.*

### Cultural desire

The data show that cultural desire appears meaningfully after engaging in honest, reflective practice and self-awareness. In addition, cultural desire facilitates the identification of cultural encounters and the need to participate in them. Thus, cultural desire allows nursing students to learn transcendently because cultural encounters are more than just clinical encounters; they help nursing students understand and enhance the complexity and genuineness of nursing care.

*Different cultures have different healthcare norms that they use and make work. It was interesting [and] educative to see how the Vietnamese healthcare system functions similarly and differently from ours* [47]*.*

*I was surprised to see that I was able to define my own cultural beliefs and values and that I often preferred my culture’s standards. I think this is due to the fact that I didn’t fully understand the deeper roots of these behaviors. It has led me to ask deeper questions about the culture* [44]*.*

### Cultural knowledge

This experiential learning process is fueled by cultural knowledge. Therefore, these themes are meaningless without relevant, realistic, and up-to-date cultural knowledge. Furthermore, this knowledge is derived not only from formal or academic sources but also from lay sources and peer learning. Therefore, the themes mentioned above are responsible for generating a conscious need for nursing students to expand their cultural knowledge.

*This experience was as challenging as it was rewarding, emboldening me to continue to explore the blatant failures of Australia’s healthcare system, seeking ways in which health professionals can move past the rhetoric to proactively work towards achieving better outcomes. Moving forward, I believe this will be my primary motivator, and be the start of what I hope to be a continued impact on the minds of my colleagues* [49]*.*

*As nurses we should be asking the questions that enable us to find out what someone believes in and what someone expects from us based on their culture. We put our own views aside in order to provide care to them. Human being to human being* [59]*.*

*It is imperative that every student experience intercultural learning, especially as we are progressing to a multicultural society. It helps us to think about differences in healthcare and gain insights about different cultures* [60]*.*

## Discussion

This metasynthesis shows that nursing students learn to become competent transcultural nurses through the combination and integration of six elements: self-awareness, reflective thinking, desire, knowledge, skills, encounters and competence. These elements are part of a multidimensional process that begins unconsciously as soon as the educational activities are offered to the students. The concepts of transcultural care and learning, together with the development of skills and attitudes, are part of a complex, continuous and evolving process. Since the first conceptualisation of the transcultural nursing model by Leininger in the 1950s, focusing on the generation of knowledge related to the care of people from different cultures [1], numerous articles have been published developing, adapting or deepening the same conceptual line [70–72]. This illustrates the changing and adaptive nature of the transcultural nursing model as a continuous process of change. Campinha-Bacote [7] addressed the different dimensions of the transcultural nursing learning process and defined the nursing model as a continuous process that integrates cultural awareness, knowledge, skills, encounters and aspirations. Purnell’s model of cultural competence is applicable to all health care providers and relates characteristics of culture to promote congruence and facilitate the delivery of consciously sensitive and competent health care [8]. Papadopoulos [73] identified four elements for the development of the cultural practice model: cultural awareness, cultural competence, cultural knowledge and cultural sensitivity. Accordingly, all these elements previously identified in the literature are present in this methasynthesis, all guided by reflective thinking.

Nursing students' personal accounts illustrate that self-awareness is key to recognising and respecting cultural practices, preferences, and diversities. It also involves understanding the philosophical aspects of nursing practice and personal life. According to the experiences of students, adopting transcultural nursing necessitates exploring intricate queries such as one's identity and purpose, as well as the concept of care. In this way, self-awareness helped the participants to recognise the fundamental components that make up people's health and the impact of current inequalities [74]. Cultural awareness, as Papadopoulos [73] notes, refers to one's understanding of their cultural identity and context. A similar definition is provided by Campinha Bacote [7], who describes it as "the examination and exploration of one's cultural and professional context" (p. 182). This involves recognizing prejudices, assumptions, and biases towards individuals who are different. Along similar lines, this metasynthesis suggests that students identify and change their negative attitudes through self-awareness. In this regard, Purnell [75] stated that increasing one's awareness of cultural diversity improves the potential for healthcare practitioners to provide culturally competent care, resulting in better care. Lack of awareness of these issues may lead to cultural imposition and the perpetuation of negative attitudes towards diversity. Therefore, nursing strategies for diverse cultural realities should be developed to steer clear of oversimplification and perpetuating stereotypes, which may not improve interactions with varied populations and even cause misunderstandings, misinformation and harm to their well-being [76]. Thus, self-awareness is a process that demands reflexive thinking to activate. This is crucial because it enables individuals to gain perspective and embrace reality which transforms their learning experience into a transformative one. Nilson [73] has stressed the significance of reflexivity in developing cultural competence and self-awareness. Therefore, reflective thinking forms the foundation of learning in transcultural nursing. To search for self-identity and self-awareness, a reflective attitude and strategies are necessary, which enable nursing students to scrutinise their thoughts, emotions, values, actions, and biases [77, 78]. Thus, reflective thinking increases self-awareness, suggesting that questioning personal feelings and values will allow nursing students to implement theoretical and practical knowledge for culturally competent care.

According to Campinha Bacote [7], a cultural encounter is the process by which professionals interact with people from diverse backgrounds. The study's results primarily focus on students' involvement in training through the adoption of innovative learning techniques. In fact, the literature suggests the use of modern approaches, including online classes [75], simulations [79], and role-playing [31, 66], to enhance the learning experience. These methodologies could facilitate the development of culturally competent care and qualities intrinsically related to transcultural nursing, such as "cultural humility". This term refers to the process of becoming mindful of how culture impacts the health of individuals and behaviours, in order to develop a careful approach to care [13, 78, 79]. Therefore, cultural encounters help nursing students to appreciate and value the various cultural differences while providing closer and more accurate care.

Cultural skills refer to the ability to provide effective care that takes into account patients' beliefs, behaviours and needs [73]. As illustrated in this metasynthesis, cultural skills cannot stand alone; to be useful, cultural skills must be acquired through the interaction of the different elements of transcultural learning. Only then will it be possible for nursing students to identify patients' actual needs and subsequently provide culturally tailored treatment plans and person-centred care. Patient-centred and holistic care, known as humanised care, has been identified as a paradigm shift [80, 81]. Despite its application in clinical practice being complex and interrelated [82], it is vital to establish effective communication with all those served [72]. However, as noted above, stereotypes and prejudices remain prevalent, although they can be reduced through cultural understanding [75] and compassion [73]. Papadopoulos and Pezzella [83] emphasise the importance of cultural competence and compassion, which refers to the human ability to understand another's distress and the desire to provide culturally appropriate health care/nursing interventions; this quality is not innate but can be developed through appropriate means. This metasynthesis indicates that cultural abilities gained through transcultural nursing learning experiences are imperative for nursing students to create personalised, effective and significant care plans. This promotes improved health outcomes by acknowledging the dignity, individuality and uniqueness of those being served, and supporting patients and practitioners in adopting compassionate care.

The development of cultural desire is not an isolated act, as it does not occur spontaneously. As shown in this metasynthesis, for nursing students cultural desire is an opportunity, a trigger for significant learning and it becomes realistic when they participate in the care process, especially through self-awareness and reflective thinking. Cultural desire is a concept that influences perceived quality and is directly related to motivation and job satisfaction [84]. This concept is similar to the definition of "cultural sensitivity" proposed by Papadopoulos [73], which refers to how professionals view the patients they treat and emphasises the development of appropriate interpersonal relationships with patients. Similarly, Campinha Bacote [7] defines cultural desire as "the motivation of the professional to want to engage in all the processes that make up the model, rather than having to" (p. 182). In the same way, this metasynthesis suggests that cultural desire emerges as an opportunity for nursing students to learn in an intangible way because cultural desire translates them into a better understanding of the complexities of nursing. The usefulness of cultural desire depends heavily on achieving cultural humility and rejecting ethnocentrism, the idea that one's own culture is the gold standard against which other cultural practices are judged [85]. In this regard, Leininger [1] stated that awareness and sensitivity to the differences and similarities in cultural care is the first step in the quest for true transcultural care (stage 1). Next, gaining knowledge (phase 2), using evidence (phase 3) and conducting research (phase 4) were needed to promote relevant transcultural care. This metasynthesis shows that cultural desire is needed throughout the process, especially in phases 2–4; cultural desire appears as the energy, a source of motivation, needed to change nursing practice and to achieve a deeper understanding of what diversity and nursing are.

Lastly, current, relevant, and realistic cultural knowledge is fundamental and, along with reflective thinking, underpins the entire learning process. For Campinha Bacote [7], this knowledge is nothing more than "the process of seeking and obtaining a solid educational base on cultural diversity and ethnic groups" (p. 182). According to Papadopoulos [73], it is important to have contact with people from different ethnic groups, referred to as "cultural encounters" in this study. This knowledge comes from formal education, lay knowledge, and peer interactions. In Europe and some parts of the world, the Erasmus + program allows for the combination of formal education with direct contact with diverse cultural experiences within the university environment, which is a unique learning opportunity for nursing students [86]. As previously stated, it is critical to highlight that the learning process of transcultural nursing does not occur spontaneously in the student; however, it requires commitment and a conscious attitude on the part of the students. Hence, knowledge alone does not lead to behavioral changes in clinical practice [87]. This component is key to understanding the relevance of providing standardized training opportunities that evolve toward meaningful learning and developing evaluation tools that allow the outcomes of these programs to be systematically analyzed [22]. Therefore, it should be committed to promoting research and cultural importance in the nursing curriculum and teaching plans [88–91]. It is especially important to remember that transcultural training in European faculties is still highly variable [92]. However, it is critical to analyze and expand existing evidence on transculturality in health and its implications in clinical practice so that the learning methods used are validated, and significant results are obtained so that well-intentioned but generalist training actions with little validity and questionable results do not occur [93].

### Limitations

This metasynthesis had some limitations that must be considered. First, despite conducting the search in English, Portuguese, and Spanish, only qualitative articles published in English were ultimately included after critical appraisal. This may have resulted in overlooking diverse experiences and perpetuating an ethnocentric approach. Secondly, there was considerable variation in the keywords used to index the articles within the databases, which posed difficulties in finding and accessing the evidence. Therefore, some studies may have been disregarded as a consequence. Moreover, due to financial and time constraints, only two databases were used. Although evidence indicates that a minimum of three databases should be used when conducting systematic reviews [94], considering Pubmed and Scopus are the largest and most popular databases, it was believed that the potential loss of articles would be minimal. Finally, although mixed-methods studies may not produce rich and meaningful qualitative findings from an epistemological and methodological perspective, and it may be risky to separate their findings, qualitative findings from mixed-methods studies were included. This decision was made due to the lack of robust qualitative studies available and as they provide deep and detailed narrative accounts. Despite the limitations mentioned, this metasynthesis has some strengths that must be considered too. The diversity of the academic profiles of the team of reviewers/researchers provides a broad, heterogeneous and critical perspective. This allows for a deeper understanding of the phenomenon under study. Similarly, the process of search, selection, essential reading and analysis was accompanied by constant contact between the main researcher and the rest of the team, always involving reflective thinking.

## Conclusions

In conclusion, transcultural nursing learning is a complex and ongoing process. It is constantly evolving, with multiple dimensions interacting: self-awareness, reflective thinking, cultural encounters, cultural competencies, cultural desires, and cultural knowledge. All of these require a thorough analysis and the development of specific skills that allow transcultural practices to be integrated into the daily lives of health professionals.

To promote the adoption of significant transcultural learning, active learning methods that allow all the elements identified in this metasynthesis to be worked on should be promoted. The use of this model in professional practice does not occur spontaneously; however, educational strategies should be implemented to encourage changes in behavior, attitudes, and reflective thinking.

## Data Availability

The datasets used and/or analyzed during the current study are available from the corresponding author on reasonable request.
